# Other-oriented perfectionism in adolescents: differences in internalizing and externalizing problems, as well as in prosocial behavior

**DOI:** 10.3389/fpsyg.2025.1580642

**Published:** 2025-04-30

**Authors:** Andrea Fuster, María Pérez-Marco, María Vicent

**Affiliations:** Department of Developmental Psychology and Teaching, University of Alicante, Alicante, Spain

**Keywords:** other-oriented perfectionism, internalizing problems, externalizing problems, prosocial behavior, adolescents, logistic regressions

## Abstract

**Introduction:**

The scientific evidence has demonstrated that other-oriented perfectionism is negatively related to internalizing and externalizing problems only in adults. However, there is a disagreement about how this perfectionistic dimension is associated with prosocial behavior in adults and adolescents. Moreover, there is a lack of knowledge about how other-oriented perfectionism is associated with those variables in youth population. For this reason, the aims of this study were to: (1) examine differences between students with high and low scores on internalizing and externalizing problems, and prosocial behavior based on other-oriented perfectionism; and (2) determine the likelihood of exhibiting high levels of these indexes based on perfectionism scores.

**Method:**

681 students aged 12 to 16 (*M* = 14.14, *SD* = 1.31), completed the Other-Oriented Perfectionism Subscale-Junior Form and the Strengths & Difficulties Questionnaire.

**Results:**

The three indexes (i.e., internalizing problems, externalizing problems, and prosocial behavior) were calculated as the mean of the subscales that composed each index. After obtaining the total scores of internalizing problems, externalizing problems, and prosocial behavior, the scores of each were dichotomized into high and low scores. Student's t-test reported significant differences between students with high and low scores on the three indexes based on the other-oriented perfectionism dimension. Furthermore, the logistic regression analysis reported that the probability of exhibiting a high level of internalizing and externalizing problems increases for each point increase in other-oriented perfectionism. Contrarily, the likelihood of displaying a high index of prosocial behavior decreases for each point increase in this perfectionistic dimension.

**Discussion:**

The results suggest the maladaptive role of other-oriented perfectionism, underscoring the need for further research on how this perfectionistic dimension impacts the educational environment.

## 1 Introduction

Perfectionism is a multidimensional and complex personality trait (Flett and Hewitt, 2020). Distinct models have established theories regarding this construct, such as that of Hewitt and Flett (1991), which proposes three perfectionistic dimensions: socially prescribed perfectionism (SPP), referring to the beliefs regarding the demands imposed by the environment for the individual to achieve perfection; self-oriented perfectionism (SOP), referring to the self-imposition of high standards, as well as self-criticism and strivings for perfection; and other-oriented perfectionism (OOP), described as the tendency to demand perfectionism from others.

Based on this model, Flett et al. (2016) created the Child and Adolescent Perfectionism Scale (CAPS) to assess perfectionism with the SPP and SOP dimensions. More recently, Hewitt et al. (2022) developed a specific scale to assess the OOP dimension in children, given that Hewitt et al. (2017) provided an in-depth theoretical account of how OOP could emerge during childhood. Therefore, although the SPP and SOP dimensions have already been extensively studied in the juvenile population using the CAPS (an instrument that is designed for this age range), evidence of OOP in children and adolescents is scarce. This evidence has been traditionally assessed using the Hewitt Multidimensional Perfectionism Scale, which was designed for adults (HMPS, Hewitt et al., 1991) (e.g., Damian et al., 2022) or the Social Perfectionism Questionnaire (Oros et al., 2019) (e.g., Chemisquy and Oros, 2020). However, its study during childhood is also relevant. Flett and Hewitt (2020) claim that perfectionists with OOP appear to be a real issue because they tend to control and make demands of others. Furthermore, these authors report this kind of perfectionists also tend to be predisposed to experience and express frustration when others fail or do not exert sufficient effort which may result in social disconnection and isolation.

Internalizing problems are a heterogenous set of emotional disturbances, including anxiety, depression, withdrawal or somatic complaints (Alarcón-Parco and Bárrig-Jó, 2015). About other-oriented perfectionism and internalizing problems, previous knowledge reveals that OOP is positively associated with emotional control and emotional sensitivity (Flett et al., 1996), as well as health problems such as somatic complaints, cold/fever/nausea, or gastrointestinal issues (Saboonchi and Lundh, 2003). However, the association was only found to be significant for emotional sensitivity. Furthermore, the last authors mentioned performed multiple regression analyses, revealing that OOP was a negative and significant predictor of somatic complaints.

Specifically, regarding withdrawal, OOP has been significantly associated with displaying less fear of being alone and having an affiliative humor style in a positive and negative sense, respectively (Blankstein et al., 1993; Stoeber, 2015). Similarly, Flett et al. (1996) reported a negative and non-significant correlation between OOP and loneliness. For their part, Shafiq et al. (2023) found that OOP correlated significantly in a positive and negative sense with mattering and loneliness, respectively. They also reported that OOP correlated negatively and insignificantly with relationship with friends. Using hierarchical multiple regression analysis, authors found that OOP positively and significantly predicted loneliness. In addition, Visvalingam et al. (2024) reported that OOP was significantly and negatively associated with rejection sensitivity. Nevertheless, they also revealed that the relationship between OOP and loneliness was not significant.

For its part, externalizing problems are composed by a set of disruptive behaviors (i.e., aggressive behavior, disobedient behavior, delinquent behavior), inattention, hyperactivity and impulsivity (Alarcón-Parco and Bárrig-Jó, 2015; Goodman, 1997). The OOP is characterized as being positively associated with conduct problems in a significant (i.e., competitive social values, relationship conflict, task conflict, etc.) (Kleszewski and Otto, 2020; Stoeber, 2015) or insignificant way (i.e., social control) (Flett et al., 1996). Furthermore, Hill et al. (1997) and Stoeber et al. (2021) suggested that other-oriented perfectionists tend to be dominant and vindictive. OOP stands out in that it correlates with hostility and aggressive traits (e.g., Stoeber and Hadjivassiliou, 2022; Stoeber, 2015; Stoeber et al., 2017; Visvalingam et al., 2024).

In particular, Stoeber (2015) found that OOP correlated positively and significantly with aggressive humor style and callous traits. These authors also performed two regression analyses, one for each scale, obtaining similar results. For their part, Stoeber et al. (2017) found positive and significant bivariate and partial correlations between OOP, physical and verbal aggression, and anger. Moreover, a multiple regression analysis conducted by Stoeber and Hadjivassiliou (2022) reveals that OOP positively and significantly predicted aggression following unintentional provocation. More recently, Visvalingam et al. (2024) showed that OOP was significantly and positively associated with interpersonal hostility.

Ultimately, the prosocial behavior is understood as the actions that are intended to benefit others which can range from supporting individuals volunteering and helping them in need (Eisenberg and Miller, 1987). Some studies have reported negative and significant associations between OOP and social adjustment variables such as social support, prosocial value orientations, or altruism (Kleszewski and Otto, 2020; Stoeber, 2014, 2015), as well as positive and significant associations between OOP and social maladjustment variables such as narcissism or Machiavellianism (Stoeber, 2014). Moreover, Kleszewski and Otto (2020) classified 47 employees as other-oriented perfectionists based on their scores on the HMPS and using the four vignettes created by Hoffmann et al. (2015). The authors found that this profile was the least favored, obtaining the lowest mean scores on social skills. In addition, using hierarchical regression analyses, Flett et al. (1996) revealed that OOP was not a significant moderator between the dimensions of the Social Skills Inventory (Riggio, 1986) and psychosocial adjustment. Stricker et al. (2019) revealed that OOP negatively and significatively predicted agreeableness and sociality. And, for his part, Stoeber (2014) showed that OOP was a significant predictor of altruism and narcissism, in a positive and negative sense, respectively. Nevertheless, other literature has claimed that OOP is characterized as an adaptative dimension since it has been positively and significantly associated with assertiveness and social expressiveness or has been found to have a positive, but not significant, correlation with social skills in general and social sensitivity (Flett et al., 1996; Kleszewski and Otto, 2020).

Knowledge is lacking with regard to the association and predictive capacity of OOP with respect to internalizing and externalizing problems, as well as prosocial behavior in adolescents. The only two studies that have analyzed the association between OOP and related variables were published by Chemisquy and Oros (2020) and Hewitt et al. (2022). The first two authors found that OOP was positively and significantly associated with loneliness with peers and affinity to loneliness and was also a predictor of the same. The last authors showed that OOP was positively associated with depressive symptoms and social disconnection, only being significant for the last variable. The rest of the literature has been conducted on adults and has also focused on the study of independent variables (i.e., loneliness, somatic complaints, aggression, etc.), which could be categorized in the different indexes proposed by Goodman (1997): internalizing and externalizing problems, and prosocial behavior. Therefore, the aims of this study are to: (1) examine differences between students with high and low scores on internalizing and externalizing problems, and prosocial behavior based on OOP; and (2) determine the likelihood of exhibiting high levels of these indexes based on perfectionism scores.

Given the significant correlation between OOP and variables related to internalizing and externalizing problems and prosocial behavior, in a positive and negative sense, respectively (e.g., aggression, loneliness, relationship conflict, somatic complaints) (e.g., Blankstein et al., 1993; Shafiq et al., 2023; Stricker et al., 2019; Stoeber and Hadjivassiliou, 2022), the following is expected: *Hypothesis 1*. Students with high scores on internalizing problems report higher scores on OOP than students with low scores, and scores on OOP significantly and positively predict high levels of internalizing problems; *Hypothesis 2*. Students with high scores on externalizing problems report higher scores on OOP than students with low scores, and scores on OOP significantly and positively predict high levels of externalizing problems; and *Hypothesis 3*. Students with high levels of prosocial behavior report lower scores on OOP than their peers with low scores, and scores on OOP significantly and negatively predict high levels of prosocial behavior.

## 2 Method

### 2.1 Participants

The participating students were selected using a random cluster sampling process. The primary geographic area was the province of Alicante (center, north, south, east, and west). The secondary units were the high schools (one to two, randomly selected, and proportionate in each area, selecting 8 public and private institutes). Classrooms were the tertiary units, with two being randomly selected, one per course year from the 1st year to 4th year of compulsory secondary education. Following this system, the sample consisted of 681 students aged 12–16 (*M* = 14.14, *SD* = 1.31), of which 341 were boys and 331 were girls and 9 participants were considered as “others” (see Table 1). The distribution of the sample based on sex and age was homogeneous, as revealed by the Chi-squared test (χ^2^ = 15.25, *p* = 0.05).

**Table 1:** Sample distribution by sex and age.

**Gender**	**12 years**	**13 years**	**14 years**	**15 years**	**16 years**	**Total**
Boys	44 6.5%	89 13.1%	81 11.9%	60 8.8%	67 9.8%	341 50.1%
Girls	38 5.6%	70 10.3%	73 10.7%	78 11.5%	72 10.6%	331 48.6%
Others	0 0.0%	1 0.1%	6 0.9%	1 0.1%	1 0.1%	9 1.3%
Total	82 12.0%	160 23.5%	160 23.5%	139 20.4%	140 20.6%	681 100.0%

### 2.2 Instruments

The *Other-Oriented Perfectionism Subscale-Junior Form* (Hewitt et al., 2022; Fuster et al., in press). It consists of 10 items that assess the OOP dimension proposed by Hewitt and Flett (1991). This dimension is defined as beliefs and expectations about the capabilities of others (e.g., “I need my family members to be perfect”). The scale is completed using a Likert scale (1 = not at all; 5 = extremely) and its level of reliability for this study was α = 0.91.

The *Strengths and Difficulties Questionnaire* (SDQ; Goodman, 1997; Rodríguez-Hernández et al., 2013). It consists of 25 items divided into 5 scales: (I) Emotional problems (items 3, 8, 13, 16, 24; e.g., “I get a lot of headaches”); (II) Conduct problems (items 5, 7, 12, 18, 22; e.g., “I usually do as I am told”); (III) Hyperactivity (items 2, 10, 15, 21, 25; e.g., “I am restless”); (IV) Peer problems (items 6, 11, 14, 19, 23; e.g., “I am usually on my own”); (V) Prosocial behavior (items 1, 4, 9, 17, 20; e.g., “I try to be nice to other people”). The questionnaire is completed using a Likert scale (0 = not true; 2 = certainly true). For this study, the scores have been calculated for three indexes summing different combinations of dimensions according to Costa-Ball et al. (2023): (I) Internalizing problems (the sum of the total scores of Emotional and Peer problems dimensions); (II) Externalizing problems (the sum of the total scores of Conduct problems and Hyperactivity dimensions); (III) Prosocial behavior (the total score of this dimension). The reliability levels were α = 0.70 for internalizing problems, α = 0.68 for externalizing problems, and α = 0.67 for prosocial behavior.

### 2.3 Procedure

A meeting was held with the school leadership teams to inform them of the research process and objectives, inviting them to participate in this study. Written parental consent was then requested. Only adolescents whose parents reported their consent participated in the study. The rest of the students remained in the educational center doing the tasks considered pertinent by their teachers. About the instruments, they were applied anonymously and collectively during school hours. The average time for the administration of both instruments was 15 minutes.

This study was approved by the Ethics Committee of the University of Alicante (UA-2023-03-07).

### 2.4 Statistical analyzes

Bilateral correlations were calculated between OOP and internalizing and externalizing problems, as well as between OOP and prosocial behavior. The magnitudes of these correlations were interpreted according to Cohen (1988) values: small for values ranging between 0.10 and 0.29; moderate for values between 0.30 and 0.49, and large for values equal to or greater than 0.50.

The total scores for internalizing and externalizing problems and prosocial behavior were dichotomized in adolescents with high scores (equal to or above the 75th percentile) and those with low scores (equal to or below the 25th percentile). Subsequently, a Student's *t*-test was used to find differences between the mean scores on OOP reported for both groups in the three indexes. Moreover, *post hoc* tests were conducted to identify the indexes in which significant differences exist with regard to OOP. The effect size was obtained by calculating Cohen (1988) *d* index to find the magnitude of the differences found. It may be interpreted as follows: values between 0.20 and 0.49, between 0.50 and 0.79, and above 0.80 are small, moderate, and large effect sizes, respectively.

To analyze the predictive capacity of OOP on high levels of internalizing and externalizing problems and prosocial behavior, binary logistic regression was used, following the forward stepwise regression procedure based on the Wald statistic. Predictive ability was estimated using the *Odd Ratio* (*OR*) statistic and was interpreted in accordance with the criteria of Berlanga and Vilà-Baños (2014): positive prediction if *OR* > 1, and negative prediction if *OR* < 1.

All statistical analyses were performed using the IBM SPSS 22.0.

## 3 Results

### 3.1 Correlations and differences in OOP across internalizing, externalizing, and prosocial behavior

Positive and significant correlations with a small magnitude were reported between OOP and internalizing (*r* = 0.22) and externalizing problems (*r* = 0.18). The association between OOP and prosocial behavior was also significant, but in a negative sense (*r* = −0.13). The magnitude of this association was also small.

In Table 2, the data report significant differences in the levels of OOP among adolescents with high scores on internalizing problems and those with low scores. A moderate effect size was found for this difference (*d* = 0.58).

**Table 2:** Means, standard deviations, and effect sizes for OOP from low and high group scores on internalizing problems.

**Variable**	**Levene's test**		**Low scores on internalizing problems** ***N*** = **193**		**High scores on internalizing problems** ***N*** = **171**		**Statistical significance**			
	***F***	***p***	***M***	***SD***	***M***	***SD***	***t***	***d.f***.	***p***	***d***
OOP	82.31	< 0.001	1.91	3.46	5.46	8.20	−5.25	222.80	< 0.001	−0.58

OOP, other-oriented perfectionism.

Regarding externalizing problems, the data in Table 3 reveal significant differences in the levels of OOP between students with low and high scores. The effect size associated with this difference was small (*d* = 0.43).

**Table 3:** Means, standard deviations, and effect sizes for OOP from low and high group scores on externalizing problems.

**Variable**	**Levene's test**		**Low scores on externalizing problems** ***N*** = **190**		**High scores on externalizing problems** ***N*** = **212**		**Statistical significance**			
	***F***	***p***	***M***	***SD***	***M***	***SD***	***t***	***d.f***.	***p***	***d***
OOP	36.11	< 0.001	2.14	5.08	4.89	7.50	−4.33	373.12	< 0.001	−0.43

OOP, other-oriented perfectionism.

Table 4 shows that adolescents with low scores on prosocial behavior significantly differed from those with high scores in terms of OOP. The effect size associated with this difference was small (*d* = 0.26).

**Table 4:** Means, standard deviations, and effect sizes for OOP from low and high group scores on prosocial behavior.

**Variable**	**Levene's test**		**Low scores on prosocial behavior*****N*** = **121**		**High scores on prosocial behavior** ***N*** = **417**		**Statistical significance**			
	***F***	***p***	***M***	***SD***	***M***	***SD***	***t***	***d.f***.	***p***	***d***
OOP	12.80	< 0.001	4.89	7.63	3.25	5.94	2.17	164.44	0.031	0.26

OOP, other-oriented perfectionism.

### 3.2 Logistic regressions

Table 5 presents the results of the logistic regression analysis for the probability of having high scores on internalizing and externalizing problems and prosocial behavior based on OOP. The proportion of corrected cases was 59.6% for internalizing problems, 58.5% for externalizing problems, and 77.5% for prosocial behavior. Furthermore, Nagelkerke's *R*^2^ was 0.11, 0.06, and 0.02, respectively. OOP positively and significantly predicted high scores on internalizing and externalizing problems. Specifically, the probability of having high scores on internalizing and externalizing problems was 11% and 8% higher, respectively, with each point that the OOP scores increased. For its part, OOP negatively and significantly predicted high scores on prosocial behavior. Specifically, the probability of having high prosocial behavior scores was 4% lower for each point that the OOP scores increased.

**Table 5:** Binary logistic regression for the probability of scoring high scores on internalizing and externalizing problems and prosocial behavior as a function of OOP.

**Variable**		***χ^2^***	***R* ^2^**	***B***	**SE**	**Wald**	***p***	***OR***	***CI* 95%**
Internalizing problems	Correctly classified: 59.6%	31.04	0.11	0.11	0.02	22.31	< 0.001	1.11	1.06–1.16
	Constant	−0.47			0.12	14.28	0.000	0.62	< 0.001
Externalizing problems	Correctly classified: 58.5%	19.44	0.06	0.07	0.02	14.92	< 0.001	1.08	1.04–1.12
	Constant	−0.14			0.12	1.53	0.215	0.86	
Prosocial behavior	Correctly classified: 77.5%	5.60	0.02	−0.03	0.02	5.88	0.015	0.96	0.93–0.99
	Constant	1.37			0.12	128.68	< 0.001	3.97	

χ^2^, chi-squared; R^2^, Nagelkerke squared; B, regression coefficient; SE, standard error; Wald, Wald test; p, probability; OR, odds ratio; CI, confidence interval at 95%.

## 4 Discussion

The purpose of this study was to analyze the differences between students with high and low scores on internalizing and externalizing problems and prosocial behavior based on OOP, as well as to analyze the probability of having high levels on these three indexes according to the OOP scores.

Firstly, with regard to internalizing problems, students with high scores on this index reported higher scores on OOP than students with low scores, and scores on OOP significantly and positively predicted high levels of internalizing problems. Therefore, results supported *Hypothesis 1* suggesting that adolescents who develop negative emotions, somatic complaints, and social withdrawal (Merrell, 2008) tend to score higher on OOP. It is possible that teenagers develop these problems after being socially rejected as a result of the high demands that they impose on the people around them. According to the Perfectionism Social Disconnection Model (Hewitt et al., 2006, 2017), perfectionists are motivated to relate to others by the need to be appreciated and accepted by a group, as well as by the need to avoid negative evaluations and rejection. If these needs are not met, adolescents tend to become psychologically and psychically vulnerable (Oros, 2005; Saboonchi and Lundh, 2003). Therefore, it is possible that these children may prefer solitude based on the findings of Chemisquy and Oros (2020), as a self-protection strategy to avoid feeling vulnerable. Consequently, it would be a conscious isolation (Flett and Hewitt, 2020). However, the social withdrawal may be related also with the relentless pursuit of perfection due to it requires high efforts that not all adolescents consider necessary to make. Thus, it would be reasonable for students with levels of OOP to prefer being alone rather than being in class with peers that they do not have similar goals than them.

Regarding externalizing problems, the results also supported *Hypothesis 2* since students having high scores on externalizing problems reported higher scores on OOP than students with low scores, and scores on OOP significantly and positively predicted high levels of externalizing problems. Results are aligned with the previous literature that it has analyze the relationship between OOP and externalizing problems in adults (e.g., Stoeber and Hadjivassiliou, 2022; Stoeber, 2015; Stoeber et al., 2017; Visvalingam et al., 2024). In the case of perfectionistic children and adolescents, they would expect their peers to be as thorough, work as hard as they do and follow their orders to get the best grades in class. Therefore, it would be reasonable for them to generate externalizing problems because they do not tolerate others' imperfections, just as they do not accept that their peers do not achieve the set objectives (Vicent et al., 2019a). Perhaps for those reasons, they prefer to work alone instead of in groups (Vicent et al., 2019a). Consequently, it would support Hewitt et al. (2022) position on including in the Perfectionism Social Disconnection Model (Hewitt et al., 2006, 2017), particularly in cases involving children and adolescents.

About prosocial behavior, *Hypothesis 3* was confirmed since students with high levels on this index reported lower scores on OOP than their peers with low scores, and scores on OOP significantly and negatively predicted high levels of prosocial behavior. Results are consistent with some literature focused on adults which shows that this perfectionist dimension is negatively and significantly associated with social adjustment variables (Kleszewski and Otto, 2020; Stoeber, 2015, 2014) and that it positively and significantly predicts individualistic orientations and self-interest (see Stoeber, 2015 for details). Although there is limited knowledge regarding how OOP can affect prosocial behavior in children and adolescents', Vicent et al. (2019a) claim that perfectionistic students do not rejoice in the successes of others and would rather have a 7 and be the highest grade in the class than share a 10 with someone else. Thus, other-oriented perfectionists tend to establish individualistic orientations and prioritize their personal interests over collective interests. Therefore, OOP may also be categorized as an antisocial variable (Stoeber, 2015) in adolescents.

## 5 Limitations

This study has certain limitations. First, it is not possible to compare the results with other studies on children or adolescents since, to the author's knowledge, this is the first work to analyze differences in OOP scores based on internalizing and externalizing problems and prosocial behavior. It is also unique in analyzing the predictive capacity of OOP with respect to having high levels of these factors. Therefore, it would be interesting for future research lines to replicate the study in order to shed light on this perfectionist dimension and how cultural factors may affect its development. Second, the 25th and 75th percentiles were used as a criterion to identify low and high scores on a variable that follows a normal distribution. Despite this method has been employed in educational and psychological settings (e.g., Gonzálvez et al., 2021; Vicent et al., 2019b), it would be recommended to use other types of analysis as the latent profile analysis to extract homogeneous clusters characterized by a common response profile (Schmidt et al., 2021). Third, although self-reports have been widely used to assess perfectionism in youth (Leone and Wade, 2018; Vicent et al., 2019a), other methods should also be used to assess personality traits (McCrae, 2020). Thus, future investigations could design observation guides or standard interviews to assess OOP in children and adolescents to complement self-reporting measures. Finally, longitudinal studies should be carried out to further understand OOP behavior with respect to internalizing and externalizing problems and prosocial behavior during adolescence.

## 6 Conclusion and practical implications

Despite the limitations, this study offers a novel contribution to the field of personality psychology since it is the first work to clarify the differences in OOP scores between adolescents with high and low scores on internalizing and externalizing problems and prosocial behavior, while also reporting on the predictive capacity of OOP on the same. This study is also relevant given its contribution to knowledge regarding the maladaptive characteristics of OOP in adolescents (Chemisquy and Oros, 2020; Hewitt et al., 2022). On the one hand, it predicts the development of both internalizing and externalizing problems, and, on the other hand, it predicts a lower rate of prosocial behavior. Moreover, given that Curran and Hill (2022) suggest that perfectionism rates in youth are on the rise, due to neoliberalism policies promoting individualism and competitiveness, as well as the growing importance of meritocracy, these results serve to motivate ongoing research on this dimension. Adolescence is a critical period in which experiences can influence neurobiological development and the creation of an individual's personal identity (Oliva, 2004). Therefore, knowing how OOP affects variables of educational interest (i.e., school climate, leadership, or bullying, etc.) may be beneficial to teachers, families, and educational psychologists in the design and implementation of specific interventions to reduce their maladaptive characteristics. Finally, the results suggest the need to introduce cooperative learning in classrooms mainly to promote prosocial behaviors since a good school climate and awareness of how to work in groups are key factors for academic and professional success (Amsalu and Belay, 2024; Guest, 2008). Furthermore, encouraging cooperative learning may reduce the internalizing and externalizing problems resulting from the intolerance of the imperfections of others. However, for effective cooperative learning, teachers must design specific evaluation plans detailing to students the processes and results to be evaluated (e.g., knowledge, competencies, reasoning process, work habits, attitudes and values), the sequence of educational tasks that have to follow, as well as the evaluative procedures (e.g., tests, observation) and the type of evaluation should be clearly defined (diagnostic, formative or summative) to be used (Johnson and Johnson, 2014).

## Data Availability

The raw data supporting the conclusions of this article will be made available by the authors, without undue reservation.

## References

[B1] Alarcón-Parco D. Bárrig-Jó P. (2015). Internalizing and externalizing behavior in adolescents. Liberabit 21, 253–259.

[B2] Amsalu A. Belay S. (2024). Analyzing the contribution of school climate to academic achievement using structural equation modeling. Sage Open 14, 1–11. 10.1177/21582440241227271

[B3] Berlanga V. Vilà-Baños R. (2014). Cómo obtener un modelo de regresión logística binaria con SPSS. Rev. Innov. Recer. Educ. 7, 105–118.

[B4] Blankstein K. R. Flett G. L. Hewitt P. L. Eng A. (1993). Dimensions of perfectionism and irrational fears: an examination with the fear survey schedule. Person. Indiv. Dif. 15, 323–328. 10.1016/0191-8869(93)90223-P

[B5] Chemisquy S. Oros L. B. (2020). El Perfeccionismo desadaptativo como predictor de la soledad y del escaso apoyo social percibido en niños y niñas argentinos. Rev. Colomb. Psic. 29, 105–123. 10.15446/rcp.v29n2.78591

[B6] Cohen J. (1988). Statistical Power Analysis for the Behavioral Sciences. Mahwah, NJ: Erlbaum.

[B7] Costa-Ball C. D. Cracco C. Cuadro A. López-García J. J. (2023). Strengths and Difficulties Questionnaire (SDQ): an update of the literature and instrumental study with schoolchildren. Rev. Latinoameric. Psico. 55, 140–148. 10.14349/rlp.2023.v55.16

[B8] Curran T. Hill A. P. (2022). Young people's perceptions of their parents' expectations and criticism are increasing over time: implications for perfectionism. Psychol. Bull. 148, 107–128. 10.1037/bul0000347 35357848

[B9] Damian L. E. Negru-Subtirica O. Pop E. I. Stoeber J. (2022). Becoming a perfectionistic adolescent: perceived parental behaviors involved in developmental trajectories of perfectionism. Eur. J. Person. 36, 24–46. 10.1177/08902070211012902

[B10] Eisenberg N. Miller P. A. (1987). The relation of empathy to prosocial and related behaviors. Psychol. Bull. 101, 91–119. 10.1037/0033-2909.101.1.91 3562705

[B11] Flett G. L. Hewitt P. L. (2020). Reflections on three decades of research on multidimensional perfectionism: an introduction to the special issue on further advances in the assessment of perfectionism. J. Psychoeduc. Assess. 38, 3–14. 10.1177/0734282919881928

[B12] Flett G. L. Hewitt P. L. Besser A. Su C. Vaillancourt T. Boucher D.. (2016). The Child-Adolescent Perfectionism Scale: development, psychometric properties, and associations with stress, distress, and psychiatric symptoms. J. Psychoeduc. Assess. 34, 634–652. 10.1177/0734282916651381

[B13] Flett G. L. Hewitt P. L. De Rosa T. (1996). Dimensions of perfectionism, psychosocial adjustment, and social skills. Person. Indiv. Dif. 20, 143–150. 10.1016/0191-8869(95)00170-0

[B14] Fuster A. Vicent M. Pérez-Marco M. (in press). Validation of the other-oriented perfectionism subscale-junior form in Spanish adolescents. Revista de Psicología Clínica con Niños y Adolescentes. 12, 1–6.

[B15] Gonzálvez C. Martín M. Vicent M. Sanmartín R. (2021). School refusal behavior and aggression in Spanish adolescents. Front. Psychol. 12:669438. 10.3389/fpsyg.2021.669438 33995227 PMC8117223

[B16] Goodman R. (1997). The Strengths and Difficulties Questionnaire: a research note. J. Child Psychol. Psychiatry 38, 581–586. 10.1111/j.1469-7610.1997.tb01545.x 9255702

[B17] Guest A. M. (2008). Reconsidering teamwork: popular and local meanings for a common ideal associated with positive youth development. Youth Soc. 39, 340–361. 10.1177/0044118X06297073

[B18] Hewitt P. L. Flett G. L. (1991). Perfectionism in the self and social contexts: conceptualization, assessment, and association with psychopathology. J. Person. Soc. Psychol. 60, 456–470. 10.1037/0022-3514.60.3.456 2027080

[B19] Hewitt P. L. Flett G. L. Mikail S. F. (2017). Perfectionism: A Relational Approach to Conceptualization, Assessment, and Treatment. New York, NY: Guilford Publications.

[B20] Hewitt P. L. Flett G. L. Sherry S. B. Caelian C. (2006). “Trait perfectionism dimensions and suicide behavior,” *Cognition and Suicide. Theory, Research and Therapy*, ed. T. E. Ellis (Washington, DC: American Psychological Association), 215–235. 10.1037/11377-010

[B21] Hewitt P. L. Flett G. L. Turnbull-Donovan W. Mikail S. F. (1991). The Multidimensional Perfectionism Scale: reliability, validity, and psychometric properties in psychiatric samples. Psychol. Assess. 3, 464–468. 10.1037//1040-3590.3.3.464

[B22] Hewitt P. L. Smith M. M. Flett G. L. Ko A. Kerns C. Birch S.. (2022). Other-oriented perfectionism in children and adolescents: development and validation of the Other-Oriented Perfectionism Subscale-Junior form (OOPjr). J. Psychoeduc. Assess. 40, 327–345. 10.1177/07342829211062009 35572033 PMC9092920

[B23] Hill R. W. Zrull M. C. Turlington S. (1997). Perfectionism and interpersonal problems. J. Person. Assess. 69, 81–103. 10.1207/s15327752jpa6901_5

[B24] Hoffmann A. Stoeber J. Musch J. (2015). Multidimensional perfectionism and assortative mating: a perfect date? Person. Indiv. Dif. 86, 94–100. 10.1016/j.paid.2015.06.001

[B25] Johnson D. W. Johnson R. T. (2014). La Evaluación del Aprendizaje Cooperativo. Cómo Mejorar la Evaluación Individual a Través del Grupo (A. B. Fletes Valera, Trans.). Ediciones SM.

[B26] Kleszewski E. Otto K. (2020). The perfect colleague? Multidimensional perfectionism and indicators of social disconnection in the workplace. Person. Indiv. Dif. 162:110016. 10.1016/j.paid.2020.110016

[B27] Leone E. M. Wade T. D. (2018). Measuring perfectionism in children: a systematic review of the mental health literature. Eur. Child Adolesc. Psychiatry 27, 553–567. 10.1007/s00787-017-1078-8 29098468

[B28] McCrae R. R. (2020). Get a second opinion: comment on Bleidorn et al. (2019). Am. Psychol. 75, 729–730. 10.1037/amp0000626 32673018

[B29] Merrell K. W. (2008). Behavioral, Social, and Emotional Assessment of Children and Adolescents, 3rd Edn. London: Routledge.

[B30] Oliva A. (2004). La adolescencia como riesgo y oportunidad. J. Study Educ. Dev. 27, 115–122. 10.1174/02103700477290214125131292

[B31] Oros L. B. (2005). Implicaciones del perfeccionismo infantil sobre el bienestar psicológico: Orientaciones para el diagnóstico y la práctica clínica. Anal. Psicol. 21, 294–303.

[B32] Oros L. B. Serppe M. Chemisquy S. Ventura-León J. (2019). Construction of a Scale to assess Maladaptive Perfectionism in children in its social dimension. Ver. Argent. Clín. Psico. 28, 385–398.

[B33] Riggio R. E. (1986). Assessment of basic social skills. J. Person. Soc. Psychol. 51, 649–660. 10.1037/0022-3514.51.3.649

[B34] Rodríguez-Hernández P. J. Betancort M. Ramírez-Santana G. M. García R. Sanz-Álvarez E. J. De las Cuevas-Castresana C. (2013). Puntos de corte de la versión española del Cuestionario de Cualidades y Dificultades (SDQ). Ver. Psiqu. Infant.-Juv. 31, 23–29.

[B35] Saboonchi F. Lundh L. G. (2003). Perfectionism, anger, somatic health, and positive affect. Person. Ind. Dif. 35, 1585–1599. 10.1016/S0191-8869(02)00382-3

[B36] Schmidt M. N. Seddig D. Davidov E. Mørup M. Albers K. J. Bauer J. M.. (2021). Latent profile analysis of human values: what is the optimal number of clusters? Methodology 17, 127–148. 10.5964/meth.5479

[B37] Shafiq B. Ali A. Iqbal H. (2023). Perfectionism, mattering and loneliness in young adulthood of Generation-Z. Heliyon 10:e23330. 10.1016/j.heliyon.2023.e23330 38163187 PMC10756993

[B38] Stoeber J. (2014). How other-oriented perfectionism differs from self-oriented and socially prescribed perfectionism. J Psychop. Beh. Assess. 36, 329–338. 10.1007/s10862-013-9397-7

[B39] Stoeber J. (2015). How other-oriented perfectionism differs from self-oriented and socially prescribed perfectionism: further findings. J. Psychop. Beh. Assess. 37, 611–623. 10.1007/s10862-015-9485-y

[B40] Stoeber J. Hadjivassiliou A. (2022). Perfectionism and aggression following unintentional, ambiguous, and intentional provocation. Curr. Psychol. 41, 4401–4406. 10.1007/s12144-020-00940-9

[B41] Stoeber J. Noland A. B. Mawenu T. W. N. Henderson T. M. Kent D. N. P. (2017). Perfectionism, social disconnection, and interpersonal hostility: not all perfectionists don't play nicely with others. Person. Indiv. Dif. 119, 112–117. 10.1016/j.paid.2017.07.008

[B42] Stoeber J. Smith M. M. Saklofske D. H. Sherry S. B. (2021). Perfectionism and interpersonal problems revisited. Person. Indiv. Dif. 169:110106. 10.1016/j.paid.2020.110106

[B43] Stricker J. Kritzler S. Buecker S. (2019). Other-oriented perfectionism in daily life situations: an experience sampling study. Person. Indiv. Diff. 151:109490. 10.1016/j.paid.2019.06.033

[B44] Vicent M. Inglés C. J. García-Fernández J. M. (2019a). El niño Perfeccionista: Más allá de la Excelencia. Pirámide.

[B45] Vicent M. Inglés C. J. Sanmartín R. Gonzálvez C. Aparicio-Flores M. P. García-Fernández J. M.. (2019b). Clarifying the two facets of self-oriented perfectionism: influences on affect and the Big Five traits of personality in children. Anal. Psicol. 35, 280–289. 10.6018/analesps.35.2.332891

[B46] Visvalingam S. Magson N. R. Newins A. R. Norberg M. M. (2024). Going it alone: examining interpersonal sensitivity and hostility as mediators of the link between perfectionism and social disconnection. J. Person. 92, 1024–1036. 10.1111/jopy.12868 37519015

